# Sorries seem to have the harder words

**DOI:** 10.1111/bjop.12790

**Published:** 2025-05-07

**Authors:** Shiri Lev‐Ari

**Affiliations:** ^1^ Royal Holloway University of London Egham UK

**Keywords:** apologies, iconicity, signalling, sound symbolism

## Abstract

Is someone who says ‘I'm genuinely sorry’ more sorry than someone who says ‘I'm really sorry’? The studies in this paper show that people use longer words when apologizing (Study 1) and interpret apologies with longer words as more apologetic (Study 2). This is in line with signalling accounts that propose that apologizers should incur a cost (greater production effort) to indicate the sincerity of their apologies. This behaviour illustrates a type of iconicity in communication that has not been examined so far: dynamic iconicity – iconicity that is context‐dependent rather than inherent to a word's meaning (e.g. producing long words to convey effort). These studies thus have implications for our understanding of the emergence, prevalence and role of iconicity in communication.

## BACKGROUND

Imagine a colleague promised to help with a task, but cancels last minute. Would you treat their apology any differently if they said ‘*I'm really sorry*’ vs. ‘*I'm genuinely sorry*’? Although the two sentences are semantically similar, this paper argues that the second sentence is perceived as more apologetic because it requires more effort to produce. This example illustrates a type of iconicity not previously considered, *dynamic iconicity*, which is context‐dependent rather than inherent to the word. Investigating this type of iconicity has the potential to transform our understanding of the emergence, prevalence and role of iconicity in language.

The sensitivity of lexical and grammatical choice to contextual factors is well established. For instance, speakers might prefer to say *the children's voices* rather than *the voices of the children* to keep an alternating stress pattern (Shih et al., 2015). Lexical choice, grammatical choice and word order can also be influenced by a wish to maintain uniform information density (Jaeger, 2010) or provide given information before presenting new information (e.g. Arnold et al., 2000). The current studies test whether lexical choice can also be motivated by the wish to express contextual iconicity.

### Iconicity

Iconicity in language is the non‐arbitrary relationship between form and meaning. Research on iconicity tends to focus on lexical iconicity, and specifically, on how word forms relate to their meaning. For example, people associate high front vowels, such as /i/ with small size (e.g. Newman, 1933; Parise & Spence, 2012; Peña et al., 2011; Sapir, 1929). Thus, when shown a small and a large table and asked to indicate which is called *mil* and which is called *mal*, people assign *mil* to the small one and *mal* to the large one (Sapir, 1929). Furthermore, these associations are exhibited in language, and thus, across the world's languages, the word denoting *small* is more likely to contain the vowel /i/ than would be expected by chance (Blasi et al., 2016).

Several non‐mutually exclusive explanations have been provided for lexical iconicity, including learning non‐linguistic statistical correlations from the real world (e.g. small objects resonate at higher frequencies than larger objects), shared properties (e.g. the oral cavity is relatively small when producing high front vowels) and/or shared neural mechanisms for processing the two (Sidhu & Pexman, 2018).

The type of iconicity that is investigated in this paper, however, is context‐dependent and will be referred to as *dynamic iconicity*. It is not between a word's form and its meaning but between a word's form and the meaning or attitude that the speaker would like to express in that context, not necessarily via the semantics of the word. To illustrate the difference, a study of inherent lexical iconicity might examine whether the sounds in the word *effort* or its length are associated with making an effort (e.g. does /f/require effort to produce? Are 6‐letter words difficult to type?). *Dynamic iconicity*, in contrast, examines whether the form of words that are semantically unrelated to effort (e.g. *genuinely*) can signal effort, and are even produced in order to signal effort, in certain contexts. Thus, a speaker who wants to emphasize the degree to which they are sincere in their apology might say that they are *genuinely sorry* rather than *really sorry*, because the word *genuinely* is harder to produce because of its long length, and thus better expresses the effort that the speaker is willing to make, and hence, their sincerity. The iconic relation between *genuinely* and effort or apologeticness, however, is not an inherent aspect of the word's meaning and will be absent in other uses of it. Therefore, in other contexts, such as, *She was genuinely pleased to see him*, the use of the word *genuinely* over *really* is unlikely to trigger any inference about the producer's intention of signalling the effort that they are expending.

This difference between inherent and dynamic iconicity also has consequences for the emergence of the two types of iconicity. Whereas iconicity that is inherent to a word's meaning emerges when words are coined or via form adaptation as the word evolves, context‐dependent iconicity plays a role during lexical selection, as the speaker chooses between semantically similar alternatives.

The existence of iconicity beyond word‐meaning relationships has already been discussed with regard to grammar and prosody. For instance, it has been observed that more marked content is expressed in more complex morphology or syntax (Givón, 1991). Word order also tends to reflect the order of events in the world, so adverbial clauses that describe a prior event are more likely to precede the main clause than follow it whereas the reverse is true for clauses describing an event that followed the main event (Diessel, 2008). In prosody, rising intonation is often interpreted as expressing uncertainty and emphasis is achieved by exerting production effort (e.g. Gussenhoven, 2002). There is also evidence that speakers produce faster speech when describing faster versus slower events (Shintel et al., 2006). This paper suggests that lexical choice itself might be influenced by a word's form.

### Apologies and costs

Apologies provide a particularly good opportunity to study dynamic iconicity. Apologies express regret over a prior committed offence, yet they are cheap to make. That is, they require little effort and are easy to fake – someone can express regret without feeling any. Despite being considered cheap talk, research shows that apologies are effective. For example, participants who performed poorly because of errors made by a research assistant reported feeling better and exhibited reduced aggression towards the research assistant if she apologized than if she did not. This was the case even when the apology was done in private and did not influence the poor evaluation that the participants received on their performance (Ohbuchi et al., 1989). Similarly, in a repeated prisoner dilemma game, participants who were betrayed by their partner reported more positive emotions and were more likely to resume cooperation if their partner apologized (Bottom et al., 2002).

This poses the question of how apologizers manage to convince the addressee in the sincerity of their intention. One manner to make an apology appear more sincere is to make it more costly (Chaudhry & Wald, 2022; Ohtsubo & Watanabe, 2009). For example, people perceive an apology as more sincere when it is accompanied by compensation. Thus, in the repeated prisoners' dilemma game mentioned above, apologies that were accompanied by an offer to reciprocate betrayal (so have the addressee win money at their expense) increased positive emotions and cooperation even more than apologies without such offers (Bottom et al., 2002). Apologies are perceived as more sincere even when the producer incurs a cost that is not transferred to the addressee. Thus, an apology is perceived as more sincere if the transgressor paid to deliver the apology message rather than sent it for free, or if the transgressor showed up to apologize at an early class they are not enrolled in rather than apologized at the next incidental meeting with the transgressed (Ohtsubo & Watanabe, 2009). This exemplifies a more general tendency that a message is seen as more honest if it is costlier to produce, whether the cost involves expenditure of effort, time or resources (Chaudhry & Wald, 2022).

While prior studies focused on the cost that accompanied the message, it is possible that making the message itself costlier, that is, requiring more effort to produce, can also increase its perceived sincerity.

### Effort and costs in pragmatic inferencing

Prior work in pragmatics lends support to the claim that the effort one puts into communicating a message influences message interpretation. A general assumption is that both speakers and listeners aim to minimize their effort. Relevance Theory (Sperber & Wilson, 1995) further proposes that speakers produce messages that they believe are relevant, that is, that the cognitive effects that they would bring about in the listener are worth the cognitive effort of processing them. Similarly, Gricean Maxims (Grice, 1975) assume that communication is cooperative, and that, among others, speakers adhere to the maxim of manner, namely, to be clear. Deviations from the clearest formulation should thus give rise to a conversational implicature. For example, if a speaker chooses to say, ‘Miss X produced a series of sounds that corresponded closely with the score of Home Sweet Home’, instead of the simpler and clearer, ‘Miss X sang Home Sweet Home’, it suggests that the speaker intentionally avoided the simpler phrasing and should lead the listener to conclude that the speaker was not impressed by Miss x's singing (Grice, 1975).

Speakers are expected not only to be mindful of addressees' cognitive effort but to also match their interlocutors' effort in the conversation (Tantucci et al., 2022). Failing to do so is perceived as impolite. Thus, information receivers are expected to reciprocate information by resonating the provided information and provide novel information in return. Responding with silence or only providing backchannel cues is seen as impolite (Tantucci et al., 2022).

### The current study

This paper tests whether messages that require more effort to produce are perceived as more apologetic. The paper specifically focuses on word length and frequency, two properties that render a word harder to produce. Longer words require more effort to produce as they require the execution of more motor actions. Word frequency has been shown to influence production ease at both lexical retrieval and the execution of the motor actions required to produce it (e.g. Balota & Chumbley, 1985; Inhoff, 1991). Study 1 examines apologetic and non‐apologetic tweets from the same individuals on X and finds that both celebrities and private individuals use longer words in their apologies than in other communication. In Study 2, participants ranked triads of apologetic sentences with closely matched semantics but varying in length and frequency. Participants ranked the apologetic sentences with longer words as more apologetic. Word frequency did not have an effect in either study. The difference between the costs that length and frequency incur on the producer vs. the addressee is discussed later on when interpreting their differing patterns.

## STUDY 1

### Method

#### Data availability statement

The study's method and analyses have been pre‐registered at aspredicted.org (#168039). Materials and analyses scripts are available on osf: https://osf.io/meb7r/?view_only=de09bc6521144b5ea68ef9521c0d7244.

##### Dataset

Fifty apologies were extracted from social media, primarily X (formerly Twitter),^1^ 25 by celebrities and 25 by regular users. For each apologizer, ten control tweets were also extracted (for a total of 11 tweets per user). For both apologies and control tweets, only tweets that are at least 2 sentences long were extracted. The control tweets were often the preceding 10 tweets that met the length requirement, though later tweets were used if earlier ones did not meet the length criterion.^2^ Template tweets (e.g. show announcements) were avoided as these only contained practical information and repeated frequently with the same text except for a change of location name, dates, etc.

Apologies of celebrities were identified by searching websites that cover showbiz with the conjugated forms of the word ‘apologize’. The articles provided links to the apology posts. Apologies of private individuals were identified with searches within X for tweets containing the word ‘apologize’. The original pre‐registration indicated that all 50 apologies will be of celebrities. After data collection started, a worry was raised that celebrities might consult their PR team when formulating their apologies, and therefore, differences between apology and control tweets might stem from author differences. To address this, half of the sample was extracted from private individuals, to ensure results hold across both subsets.

##### Pre‐processing

All tweets were pre‐processed by removing usernames, links, hashtags, punctuation and stop words^3^ using the snowball source in the stopwords package in R (Benoit et al., 2021). The median word length (in letters) for each tweet was then calculated. In addition to the pre‐registered analysis of word length, an exploratory analysis of word frequency was conducted, as low frequency words take longer to produce in both speech (e.g. Balota & Chumbley, 1985) and typing (e.g. Inhoff, 1991). The average (log) word frequency per tweet was calculated based on Subtlex US frequencies (Brysbaert & New, 2009).

### Results

**FIGURE 1:**
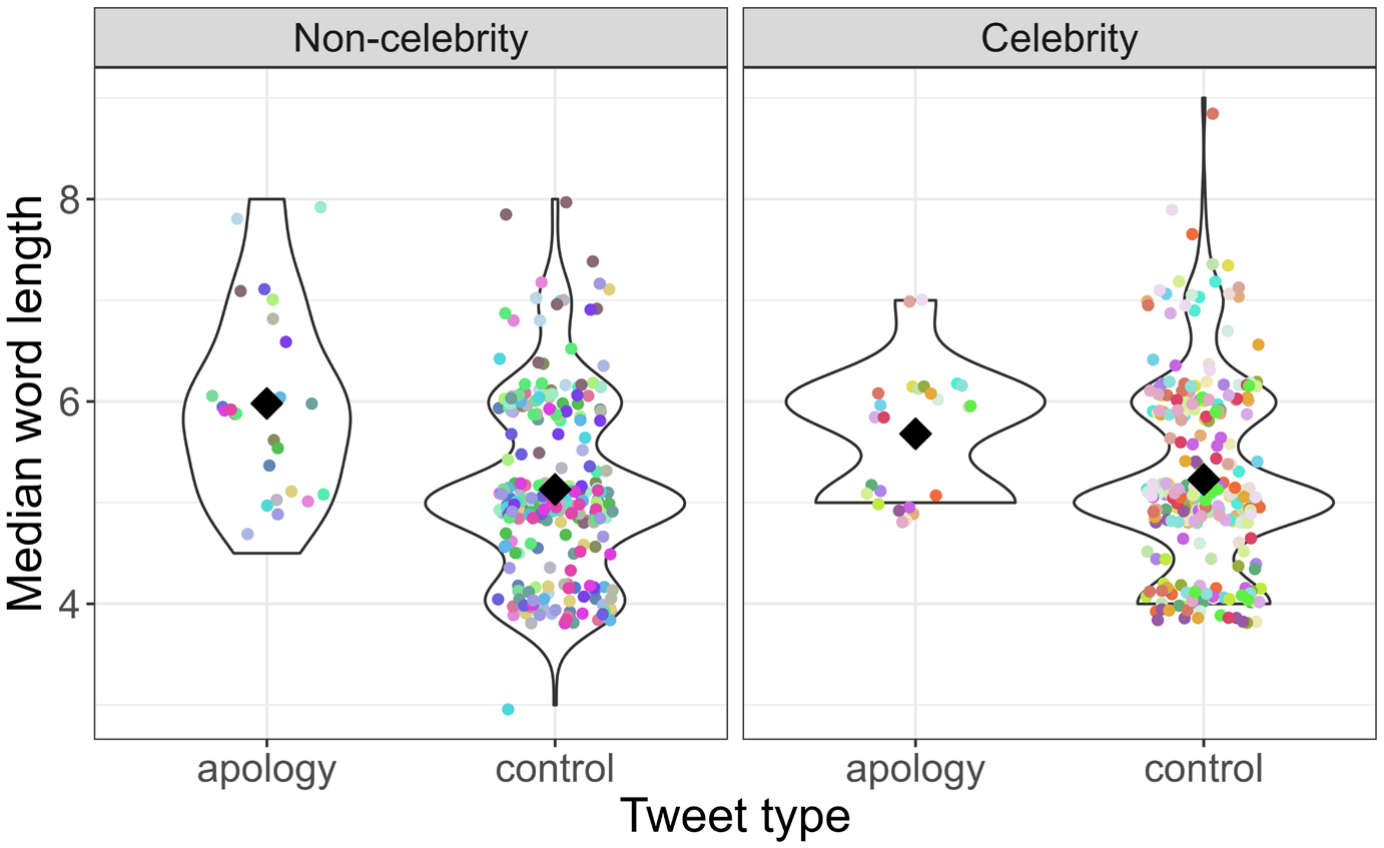
Median word length in apology and control posts by private individuals and celebrities. Each colour indicates a specific user. Black diamonds indicate condition averages.

Two mixed effects models were conducted using the lme4 package (Bates et al., 2016) in R (R Core Team, 2023), one for word length and one for word frequency. Both models included Content Type (Apology, Control), Celebrity status (Celebrity, Non‐celebrity) and their interaction as fixed effects, and Individual as a random variable. The dependent variable in the model for word length was the median number of letters per word (after removal of stopwords). The dependent measure in the word frequency analysis was the average log frequency of the words in each tweet (after removal of stopwords). Results revealed that apology tweets contained significantly longer words than control tweets (β = −0.85, SE = 0.17, *t* = −4.87; see Figure 1). Celebrity status did not influence word length (β = −0.3, SE = 0.25, *t* = −1.19) and did not interact with Content Type (β = 0.4, SE = 0.25, *t* = 1.61). The analysis of word frequency did not reveal any significant effects (Content Type: β = 0.14, SE = 0.08, *t* = 1.66; Celebrity status: β = 0.09, SE = 0.12, *t* = 0.74; Interaction: β = −0.05, SE = 0.12, *t* = −0.45).

#### Robustness checks

Apology and control posts might differ in their valence (emotional tone): Apologies are likely to be associated with negative emotions (guilt, shame, regret) and harm that was caused to others whereas control posts might express any emotion, positive or negative. To ensure that it is not valence that influences word length, the emotional tone of all posts was calculated with the sentimentr package (Rinker, 2021) and was added as a co‐variate.^4^ Emotional tone did not influence word length (β = −0.02, SE = 0.11, *t* = −0.2), and the effect of Content Type on word length remained significant (β = −0.85, SE = 0.17, *t* = −4.86).

Lastly, one may wonder whether this is a case of *dynamic* iconicity or whether the difference in word length is due to the iconicity of apology words themselves. That is, words such as *apologize* are relatively long, and it is possible that apology words themselves have evolved to be longer to convey the apologizer's effort. In that case, the findings in this paper would reflect a typical case of iconicity – an inherent relation between the form and meaning of apology words – rather than a dynamic expression of effort by choosing longer words, independent of their meaning. To examine whether the effect of word length is driven by the apology words themselves or whether apologizers used longer words in general, all apology words were removed from the apologies. This was achieved by calculating the semantic similarity of all words in apologies to the words ‘apologize’, ‘sorry’, ‘regret’ and ‘remorse’ using the gensim package in Python (Rehurek & Sojka, 2011). All words with a cosine similarity >0.7 were excluded. The excluded words were as follows: apologize, apologizing, bother, hurtful, inflamed, insulted, ok, okay, regret, remorse, sincerely, sorry, thank. An analysis on the revised dataset, without the excluded words, revealed the same effect of Content Type (β = 0.85, SE = 0.18, *t* = 4.86) and no effects or interactions with Celebrity Status. The difference in word length between the words in apology and control tweets, then, is not driven by the apology words themselves.

### Discussion

Study 1 shows that people use longer words when apologizing than in their other communication. This effect is not due to differences in valence or to the length of the apology words themselves. Instead, this effect is in line with the hypothesis that people use longer words to express the effort they are willing to exert, and thus, indicate the sincerity of their apology. The effect might seem small – words in apologies are about 1 letter longer than words in other communication. At the same time, sentences consist of multiple words, and apologies often consist of multiple sentences, so the difference in length can become substantial in longer apologies. It is worth indicating that the apology posts in this dataset did not only use longer words than non‐apology posts but were also far longer overall: apology posts by celebrities were on average 191.56 words long, whereas their non‐apology posts were on average 38.24 words long; apology posts by non‐celebrities were on average 54.4 words long whereas non‐apology posts by non‐celebrities were on average 26.92 words long. This difference in overall length potentially also signals effort on the side of the apologizers.

Interestingly, apologies did not differ from control communication in word frequency. While this analysis was not pre‐registered, low frequency words are harder to produce than high frequency words (Balota & Chumbley, 1985; Inhoff, 1991). One possibility for the null effect is that it is due to the nature of the control set ‐ users often tweet about niche topics (e.g. gaming) that are under‐represented in word frequency norms. These topic‐specific words will be highly frequent in use among the users' followers. Therefore, from the perspective of the followers, control tweets might indeed consist of higher frequency words than apology tweets but the frequency norms will not reflect this effect. Alternatively, the lack of an effect might reflect the fact that the use of low frequency words makes communication harder not only for the producer but also for the addressee (e.g. Brysbaert et al., 2018; Monsell et al., 1989), and the producer may avoid burdening the person they are apologizing to. This is in contrast to long words which are harder to produce but might not be harder to process, as longer words tend to have fewer phonological neighbours and are therefore easier to process (Meylan & Griffiths, 2024).

Overall, then, Study 1 provides evidence that language users produce longer words in their apologies than in their general communication, presumably to express the effort that they are willing to make. But is this strategy effective? Study 2 examines whether readers perceive apologies with longer and/or less frequent words as more apologetic.

## STUDY 2

To examine whether harder‐to‐produce words make apologies seem more sincere, participants read sentence triads that were closely matched in content but differed in word length and frequency. Each triad included one apology with short and high frequency words, one with short and low frequency words, and one with long and low frequency words. The experiment did not include apologies with long and high frequency words, as such words are rare. Participants ranked the apologies in terms of how apologetic they are.

### Method

#### Data availability statement

Materials and analyses scripts are available on osf: https://osf.io/meb7r/?view_only=de09bc6521144b5ea68ef9521c0d7244.

##### Participants

Fifty‐one participants, all native English speakers from the United Kingdom, were recruited via Prolific. There was no prior literature to base the sample size on, so the sample size matched the number of producers in Study 1. Two participants were excluded for failing the attention checks (see below), so all analyses were conducted with 49 participants.

##### Stimuli

Nine experimental triads were created based on apology sentences from the dataset in Study 1. ChatGPT was used to generate alternative versions without changing the meaning. Triad sentences were identical for the most part, except for replacing several words per sentence with similar words that differed in length and/or frequency (see Table 1). To further check that the versions did not differ in their semantics, cosine similarity scores were calculated using the Bert tokenizer in Scikit‐learn (Pedregosa et al., 2011). Only triads in which all paired sentences scored at least 0.88 were kept. Apology triads had an average cosine similarity of 0.94 (range: 0.88–0.97).

**TABLE 1:** Example of triad sentences. The versions are identical except for the bolded words which were selected to be short and of high frequency, short and of low frequency, or long and of long frequency, depending on condition.

Condition	Short and high frequency words	Short and low frequency words	Long and low frequency words
Example 1	I did not mean to **answer** in a **hostile way** [Average length: 5 letters; Average log frequency: 4.07]	I did not mean to **reply** in a **combative style** [Average length: 5.75 letters; Average log frequency: 2.96]	I did not mean to **respond** in a **confrontational manner** [Average length: 8.0 letters; Average log frequency: 2.90]
Example 2	I now **see** that joking about these issues is **very** rude [Average length: 4.4 letters; Average log frequency: 3.87]	I now **realize** that joking about these issues is **highly** rude [Average length: 5.33 letters; Average log frequency: 3.34]	I now **recognize** that joking about these issues is **exceedingly** rude [Average length: 6.5 letters; Average log frequency: 3.07]

Paired t‐tests were used to verify differences in word length and frequency across conditions. Apology triads were first pre‐processed to remove stopwords using the snowball source in the stopwords package in R (Benoit et al., 2021). Word length (number of letters) and log frequency (from Subtlex‐US; Brysbaert & New, 2009) were calculated for each word, and average values were computed per apology. Therefore, the length and frequency values were based both on the words shared across the versions and the ones that differed across them. Despite high overlap, apologies in the Long Low Frequency condition had significantly longer words (M = 7.77 letters) than those in the Short Low Frequency (M = 6.05 letters, *t*(8) = 12.1, *p* < .001) and Short High Frequency conditions (M = 5.45 letters, *t*(8) = 11.7, *p* < .001). The Short High Frequency and Short Low Frequency conditions did not match perfectly in word length (*t*(8) = 5.4, *p* < .001), but the difference between these conditions (0.60) was much smaller than the difference between either of these conditions and the Long Low Frequency condition (1.72 and 2.32, respectively). The conditions differed in their word (log) frequency as intended: the Short High Frequency condition had significantly higher frequency words (M = 3.76) than the Short Low Frequency condition (M = 2.87, *t*(8) = 8.5, p < .001) and the Long Low Frequency condition (M = 2.86, *t*(8) = 6.4, *p* < .001). Words in the Short Low Frequency and Long Low Frequency conditions did not differ in their frequency (*t*(8) = 0.06, *p* > .95).

Additionally, two attention check triads were created. Each attention triad included one version that was not apologetic (e.g. ‘I am not sorry that I hurt you’) and two apologies that differed in how apologetic they were (e.g. ‘I am sorry that I hurt you’ vs. ‘I am very sorry that I hurt you’).

##### Procedure

Participants were informed of the study and provided their consent for participation. Apology triads were presented in random order. For each triad, all apologies displayed on the same screen in three lines, with their internal order randomized per participant. Instructions at the top and bottom of the screen, displayed in red, guided participants to drag the most and least apologetic apologies to these locations, respectively. Participants could take as long as needed as well as change their selection before confirming by pressing ‘Next’.

### Results

**FIGURE 2:**
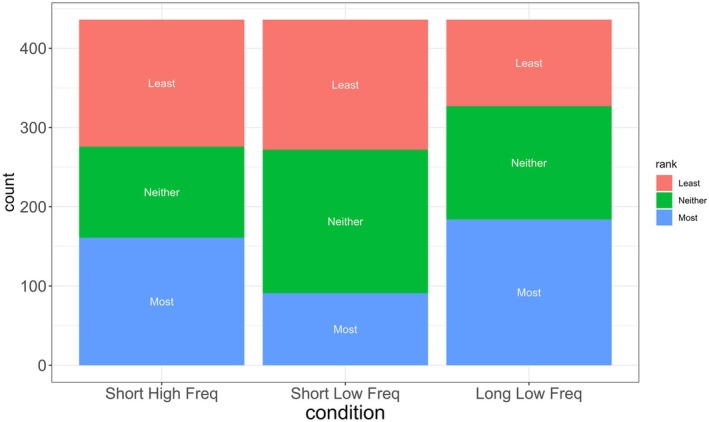
The number of items from each condition (Short and High Frequency words, Short and Low Frequency words, and Long and Low Frequency words) that were ranked as least apologetic (pink), most apologetic (blue) or neither (green).

Separate analyses were conducted to test the effects of word length and word frequency. First, rankings were calculated such that apologies that were ranked as most apologetic received a rank of 1, those that were selected as least apologetic – a rank of 3, and the remaining ones – a rank of 2. To test the effect of word length on apology perception, the difference between the ranking of the apologies with Long Low Frequency and that of apologies with Short Low Frequency words was calculated. If the apology with Long Low Frequency words was ranked as most apologetic and the apology with Short Low Frequency words was ranked as neither most nor least apologetic, the difference was 1. If the former was ranked as most apologetic and the latter as least apologetic, the difference was 2. If the apology with Short Low Frequency words was ranked as more apologetic than the apology with Long Low Frequency words, then the ranking difference was negative. An intercept‐only mixed effects model with ranking difference as the dependent variable and Participant and Item as random variables showed that the intercept was significantly above 0 (β = 0.34, SE = 0.13, *t* = 2.59) indicating that apologies with Long Low Frequency words were perceived as more apologetic than those with Short Low Frequency words (see also Figure 2). In other words, word length influences perception of how apologetic an apology is.

To examine whether word frequency influences apology perception, the difference between the ranking of the apologies with Short Low Frequency words and those with Short High Frequency words was calculated in the same manner. An intercept‐only mixed effects model with Participants and Items as random variables did not reveal any difference between the perception of these two types of apologies (β = −0.17, SE = 0.21, t = −0.82). Thus, unlike word length, there is no evidence that word frequency influences apology perception.

### Discussion

The results of Study 2 align well with those of Study 1. While Study 1 shows that individuals use longer words in their apologies, potentially to indicate the effort they are willing to exert, and therefore the sincerity of their apology, Study 2 shows that individuals correspondingly interpret word length as a cue to how apologetic the apologizer is. In contrast, neither study supports the hypothesis that word frequency plays a similar role. Individuals were not more likely to produce low‐frequency words in their apologies than in their other communications, and individuals do not perceive apologies with lower frequency words as more apologetic.

## GENERAL DISCUSSION

Languages balance iconicity and arbitrariness. Whereas iconicity facilitates language acquisition and processing (Imai et al., 2008; Kantartzis et al., 2011; Thompson et al., 2009), it is argued to be limited to concrete meanings and hinder generalization (Lupyan & Winter, 2018). Research on iconicity, however, focuses on how the form of a word is related to its general meaning, yet iconicity might take additional forms. Specifically, words' form might relate not only to their general meaning, but to the speaker's intention or attitude in context. This paper examines one specific case of such iconicity, the production of harder‐to‐produce words to express the effort that the apologizer is willing to exert.

### Iconicity beyond form‐meaning relationships

The existence of iconicity beyond word‐meaning relationships has already been examined with regards to grammar and prosody, from clause ordering according to temporal relations (Diessel, 2008) to using rising intonation to express uncertainty (e.g. Gussenhoven, 2002). This paper suggests that lexical choice can similarly be used to express context‐specific meaning. That is, while prior investigation of iconicity at the lexical level examined inherent and fixed relations between a word's form and its meaning, the studies reported here show how the choice of a word in context can express remorse, even though the word itself does not convey apology in its meaning and might not be associated with feeling remorse in its common use.

This paper, thus, opens new research directions that can shed light on the role of iconicity in communication. Understanding how dynamic iconicity can influence word choice will not only influence our understanding of the prevalence of iconicity in communication but also of its purpose and evolution. For example, iconicity has been proposed to emerge predominantly because of its facilitatory role in language acquisition, and correspondingly, it was found to be more prevalent in words acquired early (Monaghan et al., 2014). Dynamic iconicity, however, cannot facilitate lexical acquisition as the iconic relation is not with a word's meaning. Similarly, inherent form‐meaning iconicity emerges when words are coined and is honed during the process of language evolution and change, but dynamic iconicity emerges during lexical choice. Therefore, the mechanism by which dynamic iconicity arises and is maintained in the language is different from the mechanisms that give rise to inherent lexical iconicity. Interestingly, dynamic iconicity might lead to inherent lexical iconicity. For example, greater reliance on longer words in apologies might shift the meaning of the selected words to be more closely associated with apologies and remorse than their synonyms, eventually leading to semantic differentiation. Studying dynamic iconicity, then, can offer new perspectives on the emergence and spread of iconicity in communication.

### The influence of cost on interpretation

This paper shows that language users use longer words when apologizing than in their general communication, and that this strategy is effective, as people perceive apologies with longer words as more apologetic. Prior research has shown that apologies are perceived as more sincere when the apologizers demonstrate that they are willing to incur a cost (Bottom et al., 2002; Chaudhry & Wald, 2022; Ohtsubo & Watanabe, 2009). This paper suggests that modifying the effort in production, such as by using longer words, can already be effective in signalling greater remorse.

Curiously, while word length was used as an indicator of remorse, word frequency was not. From the perspective of a signalling account, we might expect word frequency and word length to play similar roles, as both influence production effort (e.g. Balota & Chumbley, 1985; Inhoff, 1991). There could be several reasons for the lack of an effect of word frequency. One possibility is that in Study 1 frequency norms under‐estimated word frequency in the control tweets because of their niche topics, and that in Study 2, the manipulation of word frequency was not strong enough. The use of word‐based frequencies rather than phrasal frequency might have also distorted the results. For example, it could be that *terribly* has much lower frequency than *very* is but that the combination *terribly sorry* will not be that much less frequent than *very sorry*. In other words, the frequency measures might have not captured frequency well enough to allow testing its effects. Another possibility is that people are less aware of the influence of word frequency on production ease than they are of the influence of word length on production ease, and therefore they use the latter, but not the former, as a cue for effort. Lastly, the difference between word length and word frequency might stem from their effect on processing ease. Longer words are harder to produce but might not be harder to process. In fact, as long words tend to come from sparser phonological neighbourhoods, they might even be easier to process (Meylan & Griffiths, 2024). In contrast, low frequency words are harder to both produce and comprehend (e.g. Brysbaert et al., 2018; Monsell et al., 1989). Therefore, while individuals who produce long words incur a cost without burdening the addressee, individuals who produce low frequency words not only incur a cost themselves but ask the addressee to engage in more costly processing. Burdening the apology recipient might seem unadvisable. Further research should examine how signalling accounts of communication interact with asymmetries between producers and addressees.

A similar avoidance of burdening the addressee has been discussed with regard to the role of indirectness in politeness.^5^ Politeness has often been associated with indirectness and wordiness in general (e.g. Brown & Levinson, 1987). Leech (2014) shows how pos‐politeness can be increased with intensifiers, and neg‐politeness can be increased with hedges and downgrades. Both types of linguistic tools often lead to longer statements. At the same, time, as Blum‐Kulka (1987) and Terkourafi (2015) have shown, indirectness is only perceived to be more polite if it is conventional. Thus, the conventionally indirect request, *Would you mind moving your car*, is perceived to be more polite than the direct request, *Move your car*, but also more polite than the even more indirect hint, *We don't want any crowding*. It has been argued that non‐conventional indirectness imposes a large cost on the addressee as it requires a lengthy inferential process. Such imposition is perceived as less polite than conventionally indirect requests (Blum‐Kulka, 1987).

### Limitations

The findings reported in this paper are novel and promising. At the same time, the studies have several limitations. Study 2 compared the perception of different versions of apologies. While there was an attempt to keep the semantics constant and only vary word length and word frequency, the words in the different versions were not perfect synonyms, and it is possible that the apologies slightly varied in meaning in a manner that influenced perception of remorse. The apologies in Study 2 were also provided without context which is likely to influence apology perception. Additionally, participants indicated how apologetic the producers were but it is unclear whether these judgements would also influence apology acceptance. Further research is required to ensure the robustness of the results and their influence on apology acceptance.

The comparison of apology tweets to all other communication might have also masked specific patterns. The goal of Study 1 was not to compare apologies to any specific type of communication but to apologizers' general communication style. That said, collapsing over different communication styles might mask important differences between the different styles. In other words, it could be that different communication styles differ in their typical word length and that not all use shorter words than apologies. That said, it is worth mentioning that Study 2 demonstrated that even within apologies, word length is interpreted as a signal of degree of remorse.

The apologies in both studies were in written form. While longer words require more effort to produce than shorter words in both spoken and written form, it is possible that the relative cost associated with them varies across modalities in a manner that influences the inferences that one draws from word length. On the one hand, the greater time pressure during synchronous spoken communication might reduce speakers' ability to adapt lexical choice to signal cost. On the other hand, production of longer words during spoken communication might be interpreted as a stronger signal of effort, as it might seem even more costly. Modality differences in processing demands might also influence addressees' ability to draw pragmatic inferences. Further research should examine whether the patterns reported in this paper extend to the spoken modality.

Lastly, all apologies, in both studies, were in English, and all participants in Study 2 were native speakers of British English. As the tendency to believe a gesture more when the actor intentionally incurs a cost seems to hold across different cultures (Bottom et al., 2002; Chaudhry & Wald, 2022; Ohtsubo & Watanabe, 2009), it seems likely that the results would generalize beyond English. Nevertheless, further research should investigate local instantiations of such patterns across different languages and cultures, as well as examine how the results generalize across different contexts, and whether they influence apology reception.

## CONCLUSION

This paper shows that individuals produce longer words in their apologies than in their non‐apologetic communication, presumably to express the effort they are willing to exert to express their remorse and/or correct the situation. Correspondingly, individuals interpret apologies with longer words as more apologetic. This might be one case of a more general phenomenon, dynamic iconicity, in which lexical choice is influenced not only by a word's meaning but also by the fit of its form with the intention or attitude that the producer would like to express. This paper, thus, opens a new direction for research on the role of iconicity in communication.

## AUTHOR CONTRIBUTIONS

**Shiri Lev‐Ari:** Conceptualization; data curation; writing – review and editing; visualization; writing – original draft; investigation; methodology; formal analysis.

## Data Availability

The data that support the findings of this study are openly available in osf at https://osf.io/meb7r/?view_only=a80d52b6d75d42588c242b30bc45499e.

## Footnotes

^1^
The apologies and control tweets from all 25 private individuals and 21 of the 25 celebrities were taken from X (twitter.com). For 4 celebrities, the apologies and control posts were taken from Instagram. There was greater focus on X because many apologies on Instagram were posted as stories that have already expired. It was also difficult to identify enough control posts on Instagram that met the length requirement (at least 2‐sentences long).

^2^
There was a preference for extracting earlier rather than later tweets, in case the apologetic tone is maintained in the tweets immediately following the apology.

^3^
Stopwords are common words in the language, such as determiners, prepositions, and pronouns. It is common to remove them before analysing texts.

^4^
Sentimentr package (Rinker, 2021) calculates the polarity of texts on a continuous scale. It treats each sentence as a bag‐of‐words and looks up each polarized word in the sentence in a sentiment dictionary. Additionally, the package takes into account valence shifters (negation, intensifiers etc.) when calculating a sentence's sentiment.

^5^
Politeness is influenced by both pragmalinguistic (linguistic form) and sociopragmatic (social context) aspects of the interaction. This paper focuses exclusively on the pragmalinguistic aspects.
